# PAMAM-Functionalized Cellulose Nanocrystals with Needle-Like Morphology for Effective Cancer Treatment

**DOI:** 10.3390/nano11071640

**Published:** 2021-06-22

**Authors:** Yanzhen Sun, Xiaoli Ma, Xiaodong Jing, Hao Hu

**Affiliations:** 1 Institute of Biomedical Materials and Engineering, College of Materials Science and Engineering, Qingdao University, Qingdao 266071, China; sunyanzhen1920@163.com (Y.S.); Jingxiaodong5230@163.com (X.J.); 2 Qingdao Institute of Measurement Technology, Qingdao 266000, China; maxiaoli1989@yeah.net

**Keywords:** cellulose nanocrystals, PAMAM dendrimer, cationic gene carrier, gene transfection, gene therapy

## Abstract

Gene therapy is used to correct or compensate for diseases caused by gene defects and abnormalities. Improving the transfection efficiency and reducing the toxicity of gene carriers are the keys to gene therapy. Similar to a typical cationic gene carrier—polyethylenimine (PEI, 25 kDa)—the polyamidoamine (PAMAM) dendrimer also has a large number of amino groups. These amino groups can be complexed with nucleic acids after protonation under physiological conditions. However, the concentrated positive charge can cause undesirable cytotoxicity. Cellulose nanocrystals (CNCs) have good biocompatibility and unique needle-like morphology, and have been proven to be efficiently taken up by cells. In this article, three-dimensional spherical PMAMA dendrimers are conjugated onto the surface of CNCs to obtain a kind of needle-like cationic carrier (CNC-PAMAM). PAMAM dendrimers act as anchors to bind the plasmid DNAs (pDNA) to the surface of the CNC. The prepared CNC-based carrier showed high transfection efficiency and low toxicity. The CNC-PAMAM can effectively deliver the suicide gene to the tumor site, enabling the suicide gene/prodrug system (cytosine deaminase/5-fluorocytosine (CD/5-FC)) to play an effective anti-tumor role in vivo. This research demonstrates that the functionalization of CNCs with PAMAM dendrimers is an effective method for developing novel gene delivery systems.

## 1. Introduction

Gene therapy can not only treat hemophilia, solid tumors, and various types of blindness; it can theoretically cure countless human genetic diseases [1]. However, safe and effective gene therapy is not an easy task. Nucleic acids are negatively charged and repel the cell membrane, resulting in poor endocytosis and endosome escape [2,3]. Furthermore, the nucleic acid is easily degraded by ribozymes in tissues or cells. Therefore, the naked nucleic acid cannot be directly administered by oral administration or injection, but a gene carrier is required [4,5]. Gene carriers can help nucleic acids overcome various barriers in blood vessels, the extracellular matrix, and cells [6,7,8]. Non-viral vectors have the advantages of low cost, simple preparation, convenient large-scale production, high safety, and unlimited length of exogenous genes [9]. Cationic gene carriers, such as cationic polymers, cationic polypeptides, and cationic liposomes, are the most studied non-viral gene carriers [10,11,12]. The loading capacity and the cytotoxicity of carriers are the two ends of the balance. Therefore, the structure of the cationic gene carrier needs to be precisely designed to balance its efficiency and toxicity.

Polyamidoamine (PAMAM) dendrimers have a branched three-dimensional structure. The high-density amino groups on the periphery of the PAMAM dendrimer can be protonated under physiological conditions. Then PAMAM dendrimers can be combined with nucleic acids through electrostatic interaction to form stable PAMAM-DNA nanoscale complexes [13]. However, the accompanying toxicity limits its efficiency. There have been a large number of reports on improving the bioavailability of the PAMAM dendrimer by modification. For example, modify the targeting ligand on PAMAM dendrimers to achieve active targeting of the lesion site [14]. PEGylation has helped mitigate some of the toxicity concerns of PAMAM dendrimers [15].

Natural polymers have significant advantages in biocompatibility and are considered to be ideal biomaterials. Cellulose is the most abundant renewable polysaccharide on earth. It is polymerized by D-glucopyranose through β-1,4-glucosidic bonds [16]. Cellulose and its derivatives have been widely used clinically [17,18]. Due to the existence of hydrogen bonds between cellulose molecules, part of the cellulose is tightly arranged in a crystalline structure. When cellulose fibers are hydrolyzed, defect-free needle-like crystalline residues are produced, which are called cellulose nanocrystals (CNCs). The diameter of CNCs is about 5–20 nm, the length is between 50 and 300 nm, and it has a huge specific surface area (up to several hundreds of m^2^/g) [19]. It has been reported that particles with large aspect ratios are more easily taken up by cells than spherical nanoparticles [20]. Therefore, the CNC may be suitable for constructing a matrix of drug or gene carriers. Xu proposed a method of grafting low molecular weight cationic polymer brushes with appropriate density onto polysaccharide molecules to prepare efficient and low toxicity gene carriers [21]. Until now, there have been few studies on the application of the CNC as a backbone to gene carriers.

**Figure 1:**
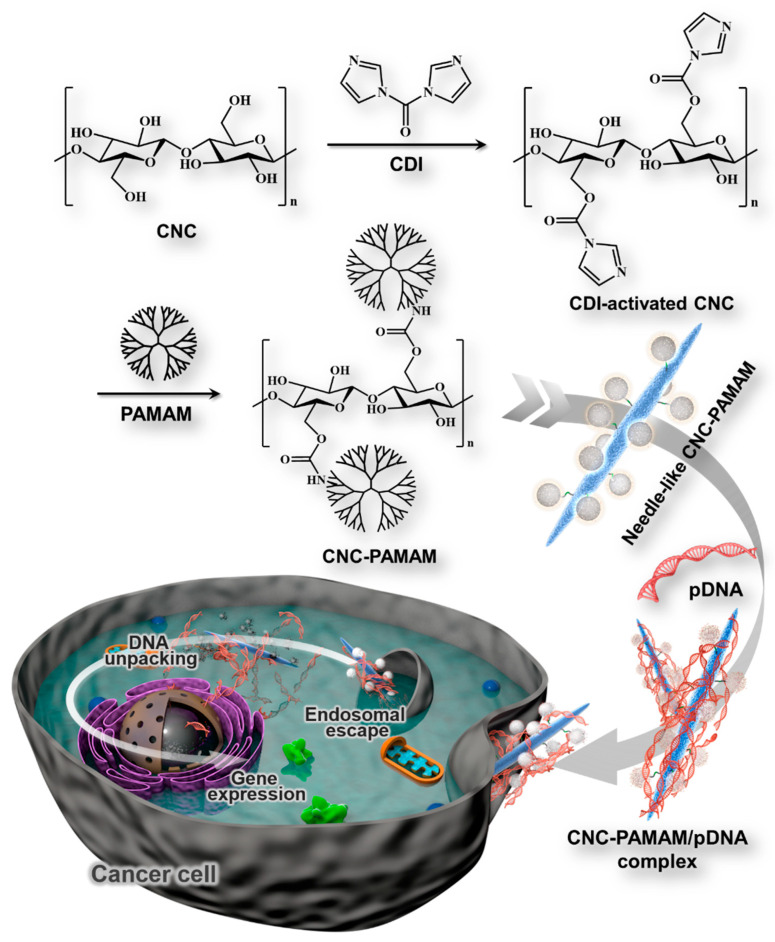
Schematic diagram illustrating the preparation of CNC-PAMAM and the resultant gene delivery process.

**Figure 2:**
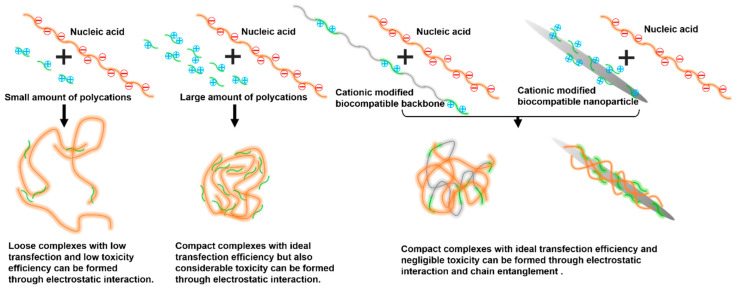
Possible structural models of the interaction between cationic compounds and nucleic acids.

In this paper, we prepared CNCs with uniform morphology by acid hydrolysis. PAMAM dendrimers of different generations were conjugated onto the CNC backbone to obtain a series of cationic gene carriers (CNC-PAMAM) (Figure 1). The gene condensation ability, cytotoxicity, gene transfection, and cellular uptake of CNC-PAMAMs were investigated in detail. The antitumor effects of the gene carriers in vitro and in vivo were evaluated by the suicide gene/prodrug system (cytosine deaminase/5-fluorocytosine (CD/5-FC)). As shown in Figure 2, we proposed structural models of the interaction between cationic compounds with nucleic acids. When the number of cationic compounds is insufficient, cationic compounds are not enough to compress nucleic acids into nanoparticles, and loose complexes with low transfection and low toxicity efficiency can be formed through weak electrostatic interaction. When the cationic compounds reach a sufficient amount, compact complexes with ideal transfection efficiency but quite high cytotoxicity can be formed. By combining the cationic compounds with a biocompatible backbone or nanoparticle, compact complexes with ideal transfection efficiency and negligible toxicity can be formed under the action of electrostatic interaction and chain entanglement. For the prepared CNC-based gene carrier, the cationic coat of PAMAM dendrimers can effectively condense with plasmid DNAs (pDNA). The low-density PAMAM scattered on the surface of the CNC may disperse the positive charge of the cationic carrier, thereby reducing toxicity. Compared with polyethylenimine (PEI, 25 kDa), that is regarded as the gold standard in transfection efficiency, cellulose has a huge specific surface area that can carry large amounts of nucleic acids, and its unique needle-like shape is more conducive to being taken up by cells. The prepared cationic gene carrier with good biocompatibility and transfection efficiency will provide a simple and effective model for cancer therapy.

## 2. Materials and Methods

### 2.1. Materials

Branched polyethylenimine (PEI, Mw ~ 25 kDa), ethylenediamine (EDA, 99.5%), methyl acrylate (MA, 99.0%), 1-ethyl-3-(3-dimethylaminopropyl) carbodiimide hydrochloride (EDAC, 98%), 1,1′-carbonyldiimidazole (CDI, 97%), 3-(4,5-dimethylthiazol-2yl)-2,5-diphenyl tetrazolium bromide (MTT), fluorescein diacetate (FDA, >98%), propidium iodinate (PI, >98%), D-mannitol (>99%), and 5-fluorocytosine (5-FC) were obtained from Sigma-Aldrich Chemical (St. Louis, MO, USA). Female Balb/c-nu mice were purchased from Institute of Laboratory Animal Sciences (ILAS). The plasmid DNAs (pDNA), including pRL-CMV encoding renilla luciferase (Promega Co., Cergy Pontoise, France), pEGFP-N1 encoding enhancedgreen fluorescent protein (EGFP) (BD Biosciences, San Jose, CA, USA), and pAdTrack-CMV-CD (pCMV-CD) encoding Escherichia coli cytosine deaminase (ECD) were amplified in Escherichia coli and purified according to the supplier’s protocol (Qiagen GmbH, Hilden, Germany). The quality and concentration of the purified pDNA were assessed by measuring its absorption at 260 nm and 280 nm, and by agarose gel electrophoresis. HepG2 and COS7 cell lines were purchased from the American Type Culture Collection (ATCC, Rockville, MD, USA).

### 2.2. Preparation of Cellulose Nanocrystals (CNCs)

CNCs were hydrolyzed by sulfuric acid as described before [10]. Briefly, the cotton wool was dispersed in sulfuric acid (64 wt%) and acidified at 45 °C for 40 min. The resulting precipitation was washed three times with deionized water and centrifuged at 10,000 rpm for 20 min. Then, it was dialyzed for at least a week to remove traces of acid. The suspension was filtered via membranes with pore sizes of 0.2 μm to remove residual aggregates prior to lyophilization.

### 2.3. Preparation of PAMAM Dendrimer

PAMAM dendrimers were synthesized by using MA and EDA as reported in our previous publication [22]. MA was added dropwise into methanol, which containing modest EDA, under a nitrogen atmosphere. The mixture was stirred at 4 °C for 30 min and an additional 24 h at room temperature. Methanol and excess MA were evaporated off using a rotary evaporator to obtain the half-generation (G0.5) PAMAM dendrimer. Subsequently, EDA was added dropwise to methanol, which contained a certain amount of G0.5 PAMAM dendrimer. The mixture was stirred at 4 °C for 30 min and then at room temperature for 24 h. After removing methanol and the excess EDA by using the rotary evaporator at 55 °C, G1 PAMAM was obtained. The steps for synthesizing high generation dendrimers are similar. The details of operation steps are available in the Supporting Information.

### 2.4. Synthesis of CNC-graft-PAMAM

CNCs (1.5 g) were dispersed in 10 mL of DMSO. CDI (0.25 g) in 2 mL of DMSO was added to the CNC suspension. The resultant mixture was continuously stirred for 24 h. Then, PAMAM dendrimers of different generation (0.2 g in 5 mL of DMSO) and triethylamine (TEA, 1.5 mL) were added to the CDI-activated CNC suspension. The mixture was reacted at 25 °C for another 24 h in a nitrogen atmosphere. When the predetermined time was reached, the reaction mixture was centrifuged three times with a mixed solution of water/MeOH (50:50, *v*/*v*). The precipitate was suspended by ultrasound and dialyzed (MWCO 3500). The end-product was freeze-dried to obtain CNC-graft-PAMAM (termed as CNC-PAMAM) (Figure 1).

### 2.5. Polymer Characterization

The chemical structure of products was determined by nuclear magnetic resonance (NMR) spectroscopy, Fourier transform infrared spectroscopy (FTIR, Perkin-Elmer Spectrum One, Perkin-Elmer Co., Norwalk, CT, USA), and X-ray photoelectron spectroscopy (XPS, Kratos AXIS HSi spectrometer, Kratos Analytical, Manchester, UK). ^1^H NMR spectra were measured on a Bruker ARX 300 MHz spectrometer (Bruker, Rheinstetten, Germany), using D_2_O as the solvent. The detailed procedures have been described in our earlier work [23].

### 2.6. Preparation of CNC-PAMAM/pDNA Complexes

The preparation of the CNC-PAMAM/pDNA complex was according to the molar ratios of nitrogen (N) in PAMAM to phosphate (P) in pDNA (or as N/P ratios). The average mass weights of 325 per phosphate group of DNA, 216 per amino group of PAMAM G3, and 225 per amino group of PAMAM G5 were assumed. All polycation/pDNA complexes were formed by mixing equal volumes of polymer and pDNA solutions to achieve the desired N/P ratio. Each mixture was kept stationary for 30 min at room temperature before being used [23].

### 2.7. Characterization of CNC-PAMAM/pDNA Complexes

The ability of various CNC-PAMAM to condense pDNA was assessed by agarose gel electrophoresis. The CNC-PAMAM/pDNA complexes at various N/P ratios were investigated. 1 μg of pDNA was used for each sample. Gel electrophoresis was performed in TAE running buffer (40 mM Tris-acetate, 1 mM EDTA) with a voltage of 110 V for 30 min using a Sub-Cell system (Bio-Rad Labs, Richmond, CA, USA), and pDNA bands were visualized and photographed using a UVP bioimaging system (BioDoc-It 220, UVP Inc., Upland, CA, USA). The particle size and zeta potential of the complexes were analyzed by dynamic light scattering (DLS) using a laser particle size and zeta potential analyzer (Malvern Nano-ZS90, Southborough, MA, USA). The sample was dispersed in deionized water to be tested.

The morphologies of the complexes were visualized by transmission electron microscopy (TEM, JEM-2100, Jeol, Japan), scanning electronic microscopy (SEM, ZEISS Sigma 300, Zeiss, Oberkochen, Germany), and atomic force microscopy (AFM, AFM5100N, Hitachi, Tokyo, Japan). TEM was operated at an acceleration voltage of 100 kV.

### 2.8. In Vitro Cytotoxicity Assay

The cytotoxicity of the prepared CNC-PAMAM was tested using a standard MTT assay. HepG2 and COS7 cells were seeded in 96-well plates at a density of 10^4^ cells/well and incubated in 100 μL of DMEM containing 10% fetal bovine serum (FBS), 100 units/mL of penicillin, and 100 μg/mL of streptomycin for 24 h at 37 °C in 5% CO_2_ inside of a humidified incubator. The culture media were replaced with fresh culture media containing serial dilutions of samples. After cultured for 24 h, the medium was discarded and 100 μL of fresh DMEM containing MTT (0.5 mg/mL) was added. After being cultured for another 4 h, the medium was removed by aspiration gently and 100 μL/well of DMSO was added. The absorbance at a wavelength of 490 nm was measured using a Bio-Rad model 680 microplate reader (Bio-Rad Labs, Richmond, CA, USA). The cell viability (%), relative to that of control cells cultured in media without polymers, was calculated from [A]_sample_/[A]_control_ × 100%, where [A]_sample_ and [A]_control_ are the absorbance values of cells treated with polymers and control cells (without the polymers), respectively. The absorbance was the average of those measured from six wells in parallel.

### 2.9. In Vitro Transfection Assay

In vitro transfection was performed with HepG2 and COS7 cell lines using plasmid pRL-CMV, which is encoded with ranilla luciferase, as the reporter gene. The cells were seeded in 24-well plates at a density of 5 × 10^4^ cells/well and incubated under standard incubator conditions for 24 h. Then, the medium was replaced with 300 μL of fresh medium (supplemented with 10% FBS) and complexes (20 μL/well containing 1.0 μg of pDNA) at various N/P ratios were added into the well. After being incubated for 4 h, the medium was aspirated, and 500 μL of fresh medium containing 10% FBS was replenished. After an additional 20 h of incubation, the cells were washed twice with PBS and, subsequently, lysed in 100 μL of cell culture lysis reagent. Luciferase gene expression was measured using a Luciferase Assay System (Promega Co., Cergy Pontoise, France). The emitted light was measured with a luminometer (Berthold Lumat LB 9507, Berthold Technology, Bad Wilbad, Germany). The protein content was analyzed using a BCA (bicinchoninic acid) Protein Assay Kit (Biorad Lab, Hercules, CA, USA). Luciferase activity was expressed as relative light units per milligram of cell protein lysate (RLU/mg protein).

The transfection ability of complexes was also visualized at their optimal N/P ratios with plasmid pEGFP (BD Biosciences, San Jose, CA, USA) in HepG2 cells. The cells were treated with samples as described above. The transfected cells were viewed under fluorescence microscope (Leica DMI3000B, Leica Microsystems GmbH, Wetzlar, Germany) using a blue filter (490 nm). Green fluorescence was observed. The percentage of the EGFP-positive cells was identified by a flow cytometer (BD LSR II, BD Biosciences, San Jose, CA, USA) [24].

### 2.10. In Vitro Antitumor Activity

FDA-PI staining was used to intuitively show the cell viability of HepG2 cells treated with 5-FC after transfection. HepG2 cells were seeded in 24-well plates at a density of 5 × 10^4^ cells/well and incubated in 500 μL of DMEM for 24 h. After discarding the culture medium, a new culture medium containing complexes at the N/P ratio of 15 was added. A quantity of 1 μg of pCMV-CD was used for each sample. After 4 h, the culture medium was replaced again with fresh medium containing 5-FC at the concentration of 40 μg/mL. After 72 h of incubation, the cells were stained with 10 μL/well of FDA (5 mg/mL in D-mannitol) and 8 μL/well of PI (2 mg/mL in D-mannitol) in darkness. Fluorescence photos were taken using a fluorescence microscope (Leica DMI3000B, Leica Microsystems GmbH, Wetzlar, Germany).

### 2.11. In Vivo Antitumor Activity

Female balb/c-nu mice, 6–8 weeks old with a weight of 18–20 g, were used in the antitumor activity test. The animal experiments were performed in accordance with the Animal Management Rules of the Ministry of Health of the People’s Republic of China (document no. 55, 2001) and were approved by the Animal Care Ethical Committee of Qingdao University (Qingdao, China). HepG2 cells in 100 μL of PBS (2 × 10^6^ cells/mL) were injected subcutaneously into the middle of the right flank of the nude mice. When the volume of the tumors reached ~50 mm^3^, the mice were divided into 4 groups (*n* = 5) randomly to evaluate the in vivo anti-tumor activity through intravenous injection every three days for a total of 6 times. In the first group, saline was intravenously injected to tumor-bearing nude mice as control (saline group). In the second group, the mice were treated with 25 μg of pure pCMV-CD (50 μL). In the third group, the mice were treated with PAMAM G5/pCMV-CD complexes containing 25 μg of pCMV-CD (50 μL, N/P ratio = 15). In the fourth group, the mice were treated with CNC-PAMAM G5/pCMV-CD complexes containing 25 μg of pCMV-CD (50 μL, N/P ratio = 15). Two days after the first injection, 5-FC (100 μg/mL) was administrated intraperitoneally at 250 mg/kg/day for 16 consecutive days. The tumor growth was monitored every two days by measuring perpendicular diameters using a caliper and the tumor volume was calculated from V = W^2^ × L/2, where W and L are the shortest and longest diameters, respectively. All mice were sacrificed 18 days later after the first injection. The tumors were dissected and imaged.

### 2.12. Statistical Analysis

Each experiment was performed at least three times, and data are shown as means ± standard deviation (mean ± SD). The statistical analysis was assessed by Student’s *t*-test. In all cases, differences were considered significant if *p* < 0.05.

## 3. Results and Discussion

### 3.1. Synthesis of the PAMAM Dendrimer

PAMAM dendrimers were synthesized by using MA and EDA as monomer pairs (Figure S1). The detailed procedures are described in the Supplementary Material. The molar ratio of the two monomers and the reaction conditions need to be precisely controlled to ensure the integrity of the dendrimer structure (Table S1). The characteristic peaks demonstrated by ^1^H NMR (Figure S2) spectra confirmed the successful preparation of the PAMAM dendrimers.

### 3.2. Preparation and Characterization of CNC-PAMAM

**Figure 3:**
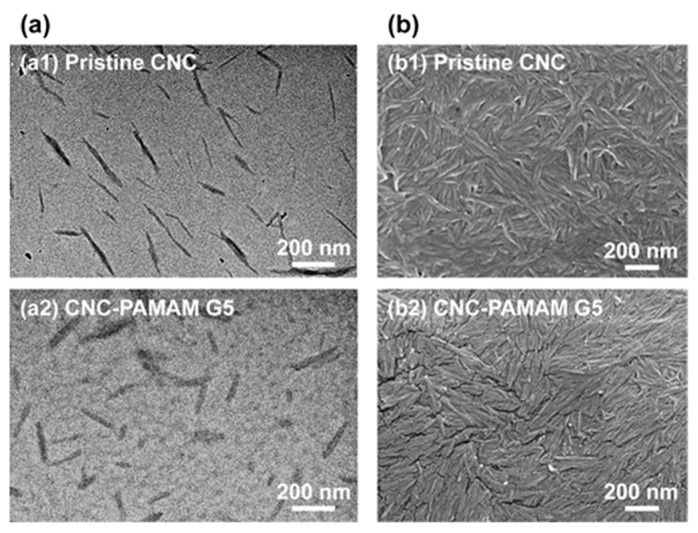
(**a**) TEM and (**b**) SEM images of the typical pristine CNC and CNC-PAMAM G5.

**Figure 4:**
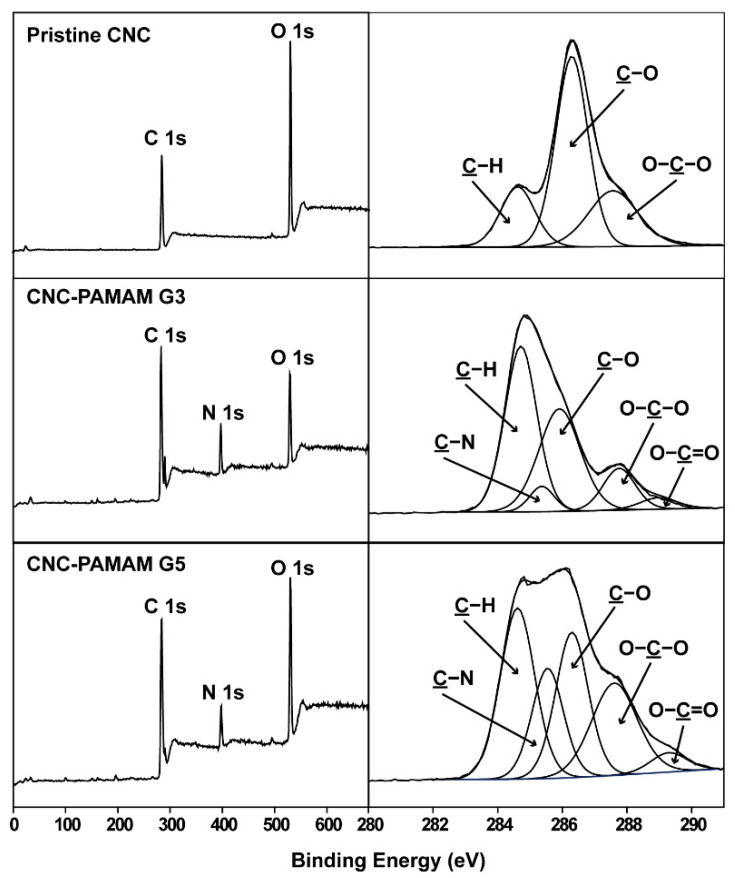
XPS wide-scan and C 1s core-level spectra of the pristine CNC, CNC-PAMAM G3, and CNC-PAMAM G5.

Through TEM (Figure 3(a1)) and SEM (Figure 3(b1)) images, it can be clearly seen that the CNC obtained by hydrolysis have a needle-like morphology with a length of about 200 nm and a width of about 15–25 nm. The numerous hydroxyl groups on CNC surfaces can be easily reacted with aminos. The third-generation PAMAM (PAMAM G3) dendrimer has a molecular weight of 6909 g/mol and has 32 amino groups on the surface, while the fifth-generation PAMAM (PAMAM G5) dendrimer has a molecular weight of 28,826 g/mol and has 128 amino groups on the surface. Under the action of the catalyst, PAMAM dendrimers are easily grafted onto the CNC surface to obtain CNC-PAMAM. The chemical structure of CNC-PAMAMs was characterized by XPS (Figure 4) and FTIR (Figure S3), respectively.

The XPS wide-scan and C 1s core-level spectra of the pristine CNC, CNC-PAMAM G3, and CNC-PAMAM G5 are shown in Figure 4. The C 1s core-level spectrum of pristine CNC can be curve-fitted by three peak components with binding energies (BEs) at about 284.6, 286.2, and 287.6 eV, attributable to the C–H, C–O, and O-C–O species, respectively [25]. In comparison with the spectrum of the CNC, a strong N 1s peak at BE of 399.8 eV appeared in the wide-scan spectra of CNC-PAMAM G3 and CNC-PAMAM G5. Their C 1s core-level spectra can be curve-fitted into five peak components with BEs at about 284.6, 285.5, 286.2, 287.6, and 288.4 eV, attributable to the C–H, C–N, C–O, O–C–O, and O=C–O species, respectively. The increase in intensities of the C-N species of CNC-PAMAM G5 was consistent with the different PAMAM contents. Figure S3 shows the FTIR spectra of the CNC-PAMAM G3 and CNC-PAMAM G5. A broad band at about 3425 cm^−1^ was assigned to stretching vibration modes of O-H groups on the backbones of CNCs. The weak peak toward 2930 cm^−1^ was attributed to the C–H antisymmetrical stretching vibration [10]. The typical absorption peaks of PAMAM dendrimers include the sharp ones at 1644 cm^−1^ (from the C=O stretch of the amide group), 1556 cm^−1^ (from C–N stretching of secondary amine), 1198 cm^−1^ (from C–N stretching of primary amine), and 1126 cm^−1^ (from C–N stretching of tertiary amine). The XPS and FTIR spectra have given evidence of the conjugation of CNC with PAMAM dendrimers. After grafting dendrimers, the profile of the CNC-based particles was still spindly (Figure 3(a2,b2)). Their diameter increased substantially and settled around a range of 25–45 nm. The substantial change of the morphologies indicates that CNCs were successfully wrapped by PAMAM dendrimers.

After collecting the samples, the masses of CNC-PAMAM G3 and CNC-PAMAM G5 are 1.556 g and 1.564 g, respectively. The dendrimers in CNC-PAMAM G3 account for about 3.6%, and the dendrimers in CNC-PAMAM G5 account for about 4.1%. According to the molecular structure of PAMAM G3 and PAMAM G5, the molar amount of nitrogen can be calculated for the subsequent calculation of the N/P ratio.

### 3.3. Characterization of CNC-PAMAM/pDNA Complexes

**Figure 5:**
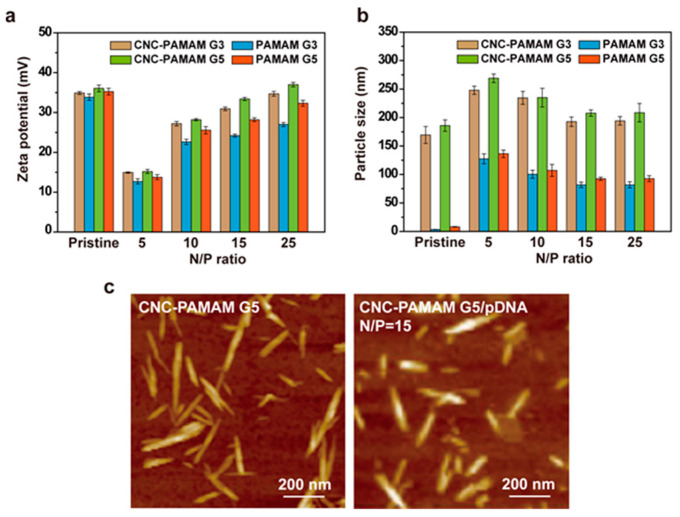
(**a**) Zeta-potential and (**b**) particle size of the CNC-PAMAM G3, CNC-PAMAM G5, PAMAM G3, and PAMAM G5 complexes compared to those of the pristine CNC-PAMAMs and PAMAMs. (**c**) AFM images of CNC-PAMAM G5 and CNC-PAMAM G5/pDNA (N/P ratio = 5).

Combining with nucleic acids and effectively forming nanoscale complexes is the first condition for gene carriers. Size, shape, and surface charge of complexes are important factors that affect cytotoxicity, cell uptake, and transfection efficiency [26]. Both the nucleic acid and the cell membrane are negatively charged, so the charge of the carrier not only affects the loading of the nucleic acid, but also affects the affinity of the carrier and the cell membrane. Zeta-potential is an indicator of complex surface charges. Zeta-potentials of CNC-PAMAM/pDNA complexes at different N/P ratios are illustrated in Figure 5a. The zeta-potential of PAMAM G3 and PAMAM G5 is around 35 mV. After PAMAM dendrimers were grafted onto the CNC surface, the surface zeta-potential of the obtained carrier is also around 35 mV. At the low N/P, due to the interaction of the negative charge of pDNA with the positive charge of the carrier, the overall zeta-potential of the carrier is low. With the increase of the amounts of carriers, the zeta-potential of the complex shows an increasing trend and finally approaches the original CNC-PAMAMs. The positively charged carriers are easy to attach to the surface of the cell membrane. On the other hand, the charged amount of the complexes also affects its cytotoxicity to cells.

Due to the incomplete blood vessels at the tumor site, nanoparticles can be enriched at the tumor site by enhanced permeability and retention (EPR) effect [27]. After PAMAM dendrimers were conjugated onto the CNC surface, the particle size of the carriers is between 160 and 200 nm (Figure 5b). It should be noted that the result can only reflect the statistics of CNC nanoparticles with different morphologies in solution because of the limitation of the DLS test principle, in which the obtained particle size is the diameter of an equivalent sphere. When the N/P is five, due to the insufficient charge on the surface of the carriers, loose complexes with larger particle size were formed between the carrier and the pDNA. With the increase of N/P, the increasing amount of positive charge brought stronger electrostatic interaction, and the pDNA was tightly compressed on the CNC surface. As shown in Figure 5b, the particle size of complexes stabilizes at about 200 nm when the N/P is greater than 15. Carriers with a particle size in this range can be enriched at the tumor site by the EPR effect. Figure 5c shows the AFM images of CNC-PAMAM G5 and CNC-PAMAM G5/pDNA (N/P = 15). It can be seen that the length of the complex remains unchanged after being condensed with pDNA, but because the surface is covered by pDNA, it becomes “slightly fat” in the radial direction. The short needle-like complexes can be efficiently taken up by cells [10,20]. The size of the nanoparticle is related to the amount of cargo carried. A small carrier is good for evading various clearance mechanisms in the body, but the cargo it carries is also limited. Enlarging nanoparticles within an appropriate range can increase the amount of cargo each carrier loaded.

**Figure 6:**
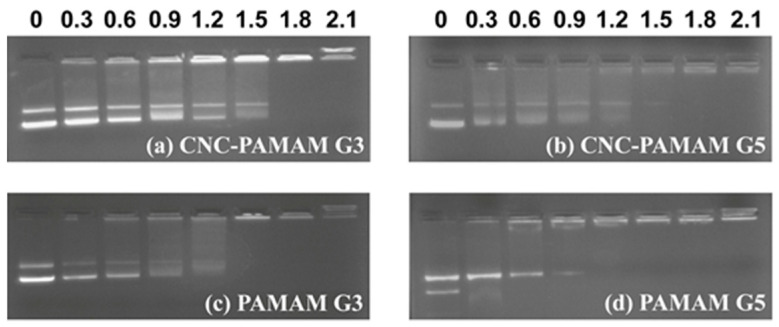
Electrophoretic mobility of pDNA with (**a**) CNC-PAMAM G3, (**b**) CNC-PAMAM G5, (**c**) PAMAM G3, and (**d**) PAMAM G5 at various N/P ratios.

The charge amount and density of the carriers have a close influence on the binding ability of the carrier to pDNA. The gel retardation results of carrier/pDNA complexes are illustrated in Figure 6. PAMAM G5 shows the strongest DNA blocking ability. All CNC-PAMAMs and PAMAMs can completely compact pDNA within the N/P ratio of 1.5. It may be that the positive charge is dispersed after PAMAM dendrimers were conjugated onto the CNC surface, so CNC-PAMAMs of the same generation have a slightly higher N/P ratio than PAMAM to block pDNA completely. In short, the prepared CNC-PMAMAs possess the essential requirements for gene carriers in terms of physical properties.

### 3.4. Cell Viability Assay

**Figure 7:**
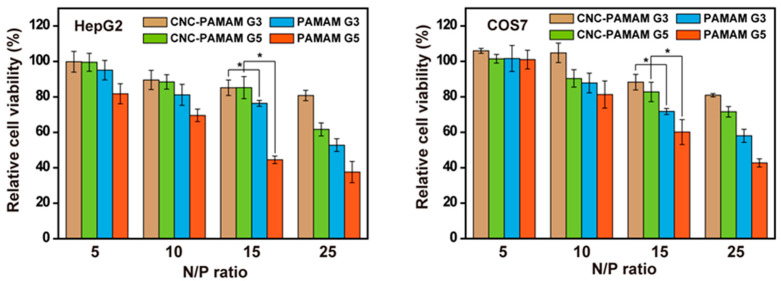
Cell viability of complexes in HepG2 and COS7 cells (mean ± SD, *n* = 6, * *p* < 0.05).

The main reason for the toxicity of the carrier is that the cation can interfere with the structure of the cell membrane, which causes the death of the cell [28]. The positive charge of cationic compounds is a double-edged sword. On the one hand, cationic compounds compress nucleic acids, protect nucleic acids from enzymatic hydrolysis, and promote cell uptake; on the other hand, cationic compounds can cause unwanted cytotoxicity. Therefore, it is important to reduce the positive charge density of cationic compounds through reasonable design and determine the reasonable concentration interval (i.e., the appropriate N/P ratio). The branched PEI with 25 kDa has long been reported to be used as a gene carrier and is regarded as the gold standard. There are a large number of amino groups in the PEI backbone, which can effectively complex with nucleic acids. But its high density of positive charge also brings undesirable toxicity. Therefore, there is a lot of research on modifying PEI [29,30]. Similarly, PAMAM dendrimers have a large number of peripheral amino groups and the number of surface amino groups can be precisely regulated by controlling the generation. We speculate that by regulating the graft density of PAMAM on the CNC surface, the charge density of the cationic carrier can be indirectly controlled. Combined with non-toxic CNC, the constructed vector may have good biocompatibility. As shown in Figure 2, the CNC serves as the backbone, while the cationic PAMAM dendrimers serve as anchor points. The nucleic acids can be tightly wrapped around the surface of the nanoparticle. The CNC has been proven to penetrate cells without showing cytotoxicity [31]. The cell viability of CNC-PAMAM/pDNA and PAMAM/pDNA complexes was evaluated in HepG2 and COS7 cells at various N/P ratios by MTT assay. As illustrated in Figure 7, with the increase of the N/P ratio, the cell viability showed a downward trend. At high N/P ratios, in addition to forming tight complexes with pDNA, a large number of free cationic compounds attacked the cell, thereby triggering cytotoxicity. At the same N/P ratio, CNC-based carriers all exhibit lower toxicity than dendrimers. This result may be due to the biocompatibility of the CNC and the positive charge of the carrier being dispersed. CNC-PAMAM G5 shows higher toxicity than CNC-PAMAM G3. This phenomenon is consistent with the fact that high molecular weight polycations exhibit higher cytotoxicity [32]. The concentrated charges of high molecular weight PAMAM dendrimers cause greater damage to cells. Overall, when the N/P ratio is less than or equal to 15, the cell viability of the cells treated with CNC-PAMAMs is higher than 80%. The introduction of CNCs improves the biocompatibility of the cationic carriers.

### 3.5. In Vitro Gene Transfection Assay

**Figure 8:**
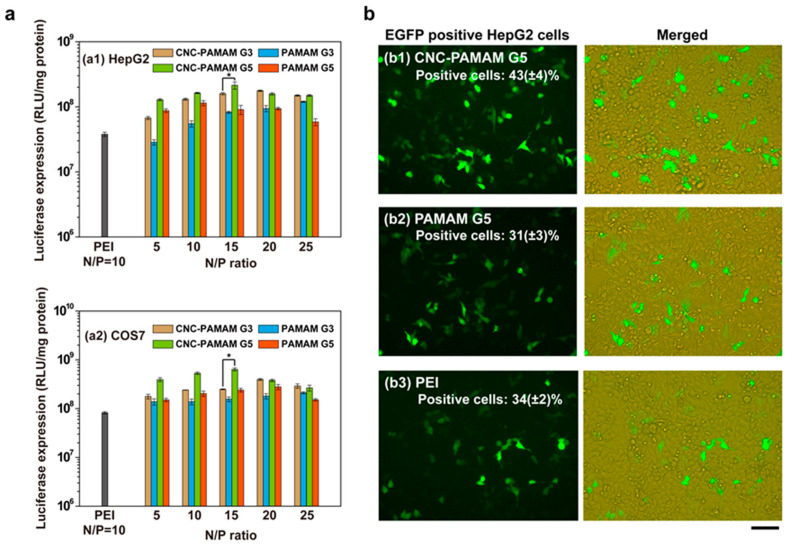
(**a**) In vitro luciferase gene expression mediated by CNC-PAMAMs and PAMAMs in comparison with those mediated by PEI in HepG2 and COS7 cells (mean ± SD, *n* = 3, * *p* < 0.05), and (**b**) representative images of pEGFP expression mediated by CNC-PAMAM G5, PAMAM G5, and PEI in HepG2 cells (scale bar: 50 μm).

Transfection efficiency is the result of many factors working together. The ideal transfection efficiency can only be achieved when the carrier has the appropriate particle size, negligible toxicity, and high loading capacity. The transfection performance of the complexes was evaluated using HepG2 and COS7 cells. Figure 8a shows the transfection efficiency of the complexes at different N/P ratios compared to those of PEI (25 kDa, at its optimal N/P ratio of 10 [33]). A trend that first rises and then remains flat or slightly declines is presented. At low N/P ratios, insufficient cationic compounds cannot effectively compress pDNA into compact nanoparticles. Such loose and large particles cannot effectively deliver DNA into the cell. When the ratio reaches 10–15, the cells treated by CNC-PAMAM G5 show the highest transfection efficiency, which is much higher than that of PEI. When the N/P ratio continues to increase, excess cationic compounds appeared in the culture medium, which brings undesired cytotoxicity. Causally, the low cell viability led to the decrease in transfection efficiency.

Regardless of the N/P ratio, CNC-PAMAMs show higher transfection efficiency than PAMAM dendrimers of the same generation. This phenomenon may be due to the good biocompatibility and appropriate aspect ratio of the CNC substrate. Our earlier work indicated that the dimensions and shape of nanoparticles have a large influence on gene transfection. The rodlike nanoparticles exhibited better gene transfection performance [34]. This result is related to the uptake efficiency of cells with different morphologies. It has been proven that cells can internalize rigid and rod-shaped nanoparticles more quickly than spherical polymer nanoparticles [20]. No matter in HepG2 cells or COS7 cells, the N/P ratio of 15 is the optimal N/P ratio of CNC-PAMAM G5. Therefore, CNC-PAMAM G5 was chosen to conduct the following experiments. The transfection performance of CNC-PAMAM G5 (N/P = 15), PAMAM G5 (N/P = 15), and PEI (N/P = 10) was observed intuitively with fluorescence microscopy in HepG2 cells. Representative images of pEGFP gene expression are shown in Figure 8b. The percentages of the EGFP-positive cells (determined by flow cytometry [24]) for CNC-PAMAM G5, PAMAM G5, and PEI were approximately 43%, 31%, and 34%, respectively. The cells treated with CNC-PAMAM G5/pEGFP complexes showed the greenest fluorescence. This indicates that most cells have effectively taken up the complex and transfected with the green fluorescent protein. The result is consistent with the luciferase expression.

### 3.6. Antitumor Effects with a CD/5-FC Suicide Gene System

**Figure 9:**
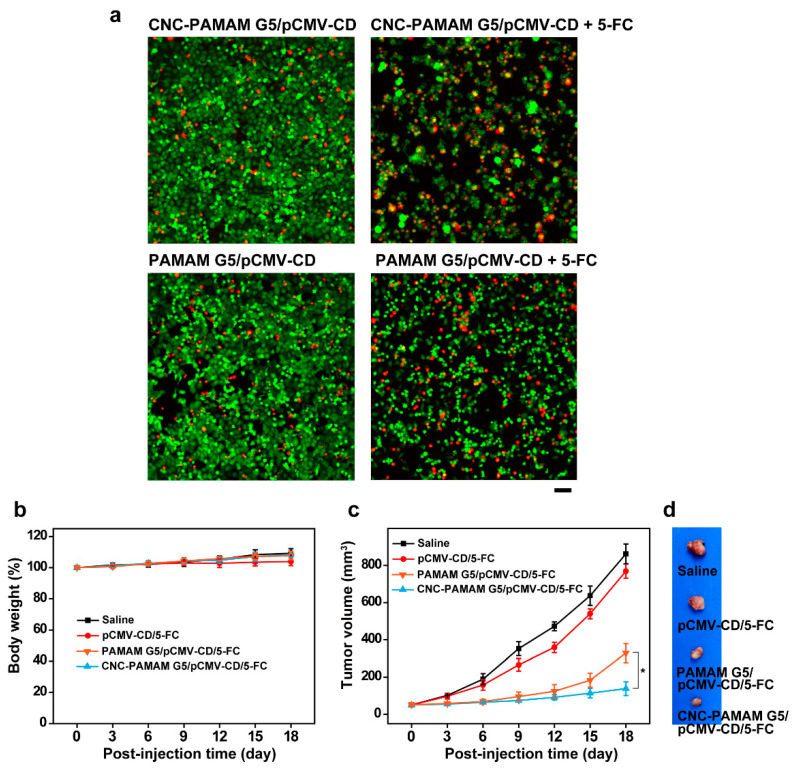
(**a**) FDA-PI staining mediated by CNC-PAMAM G5/pCMV-CD and PAMAM G5/pCMV-CD at an N/P ratio of 15 with or without 5-FC (40 μg/mL, live cells: green; dead cells: red; scale bar: 50 μm). (**b**) 18-day body weight trend and (**c**) tumor volume of the HepG2 tumor bearing nude mice administrated by the tail vein injection of saline, pCMV-CD/5-FC, PAMAM G5/pCMV-CD/5-FC, and CNC-PAMAM G5/pCMV-CD/5-FC (mean ± SD, *n* = 3, * *p* < 0.05). (**d**) Photos of dissected tumors at the 18th day post the first injection.

Liver cancer is insidious and difficult to cure. HepG2 is a perpetual cell line consisting of human liver carcinoma cells and has been widely used for scientific research. CD/5-FC is one widely investigated suicide gene/prodrug system in treating tumors [35]. pCMV-CD can express CD after transfection in cells. 5-FC has no ability to kill tumor cells. However, 5-FC can be transformed into 5-fluorouracil (5-FU) under the action of CD. 5-FU can interfere with the synthesis of DNA and RNA and is a commonly used anti-tumor drug in clinical practice. CNC-PAMAM G5 and PAMAM G5 were used to deliver pCMV-CD to HepG2 cells. The antitumor effect of cationic compounds was assessed in the presence of 5-FC (40 μg/mL). The survival of cells was observed by staining live and dead cells with FDA-PI. FDA, a nonfluorescent molecule, can hydrolyze by nonspecific esterases in viable cells to emit green fluorescence in the cytoplasm. PI, a nucleic acid binding dye, can readily enter apoptotic/dead cells and emit red fluorescence. As shown in Figure 9a, it was distinctly observed that HepG2 cells were inhibited or killed when treated with the CNC-PAMAM G5/pCMV-CD and PAMAM G5/pCMV-CD complexes in the presence of 5-FC. More red cells can be observed when the cells are treated by CNC-PAMAM G5/pCMV-CD complexes.

We further explored the delivery of pCMV-CD by CNC-PAMAM G5 and PAMAM G5 to inhibit tumor growth in vivo. During the first week, no significant difference was found in the weight change rate of nude mice in each group (Figure 9b). After six days, the tumors in the saline group and the pCMV-CD/5-FC group grew rapidly, and finally the tumor volume was 16.6 times that before treatment (Figure 9c). Mice treated with CNC-PAMAM G5/pCMV-CD and PAMAM G5/pCMV-CD complexes had much smaller tumors. Among them, the CNC-PAMAM G5/pCMV-CD group showed the best therapeutic effect (Figure 9d). The results show that the CNC-based carrier can effectively inhibit tumor growth in vivo.

## 4. Conclusions

PAMAM dendrimers were successfully conjugated to the surface of CNCs, and needle-like nanoparticles with cationic shells were obtained. The cationic shell composed of PAMAM dendrimers makes it possible for efficient gene transfection, and the needle-like CNC makes the carrier have good biocompatibility and easy for cells to take up. The physical, chemical and biological properties of the prepared CNC-based carriers were characterized in detail. Compared with PEI (25 kDa), the series of CNC-PAMAMs showed higher transfection efficiency and lower toxicity. Through the suicide gene/prodrug system test, it is found that CNC-PAMAMs have good performance in inhibiting tumor growth in vivo. In general, the prepared gene carriers developed based on CNC and PAMAM have a certain potential for gene delivery and tumor therapy.

## Data Availability

The data presented in this study are available upon request from the corresponding author.

## Supplementary Materials

The following are available online at https://www.mdpi.com/article/10.3390/nano11071640/s1, Figure S1: Reaction scheme of the PAMAM dendrimer, Table S1: The molar ratio of the reactants for PAMAM preparation, Figure S2: ^1^H NMR spectra of PAMAM G5, Figure S3: FTIR spectra of CNC, PAMAM G3, PAMAM G5, CNC-PAMAM G3 and CNC-PAMAM G5.
