# Supplementary material for: PAMAM-Functionalized Cellulose Nanocrystals with Needle-Like Morphology for Effective Cancer Treatment
Source: Nanomaterials (Basel). 2021 Jun 22;11(7):1640. doi: 10.3390/nano11071640 (PMC8307312; doi:10.3390/nano11071640)
Supplement: Supplementary file 1 [file nanomaterials-11-01640-s001.zip › nanomaterials-1232693-supplementary.pdf]

Supplementary Materials

PAMAM-Functionalized Cellulose Nanocrystals with Needle-like Morphology for Effective Cancer Treatment

Yanzhen Sun [1], Xiaoli Ma [2], Xiaodong Jing [1] and Hao Hu [1,*]

[1] Institute of Biomedical Materials and Engineering, College of Materials Science and Engineering, Qingdao University, Qingdao 266071, China; sunyanzhen1920@163.com (Y.S.); Jingxiaodong5230@163.com (X.J.)
[2] Qingdao Institute of Measurement Technology, Qingdao 266000, China; maxiaoli1989@yeah.net
* Correspondence: huhao@qdu.edu.cn

EDA was added into 25 mL of methanol, and then MA was added dropwise into the solution under a nitrogen atmosphere. The reaction mixture was stirred at 4 °C for 30 min and an additional 24 h at room temperature. Methanol and excess MA were evaporated off using a rotary evaporator. Then the half generation (G0.5) PAMAM dendrimer was obtained. To prepare PAMAM G1, a certain amount of EDA was added dropwise to 25 mL of PAMAM G0.5 dendrimer solution in methanol under a nitrogen atmosphere, and the reaction mixture was stirred at 4 °C for 30 min and then at room temperature for 24 h. PAMAM G1 was obtained after removing methanol and the excess EDA by using the rotary evaporator at 55 °C. The steps for synthesizing high generation dendrimers are similar and the molar ratio of the reactants was summarized in Table S1.

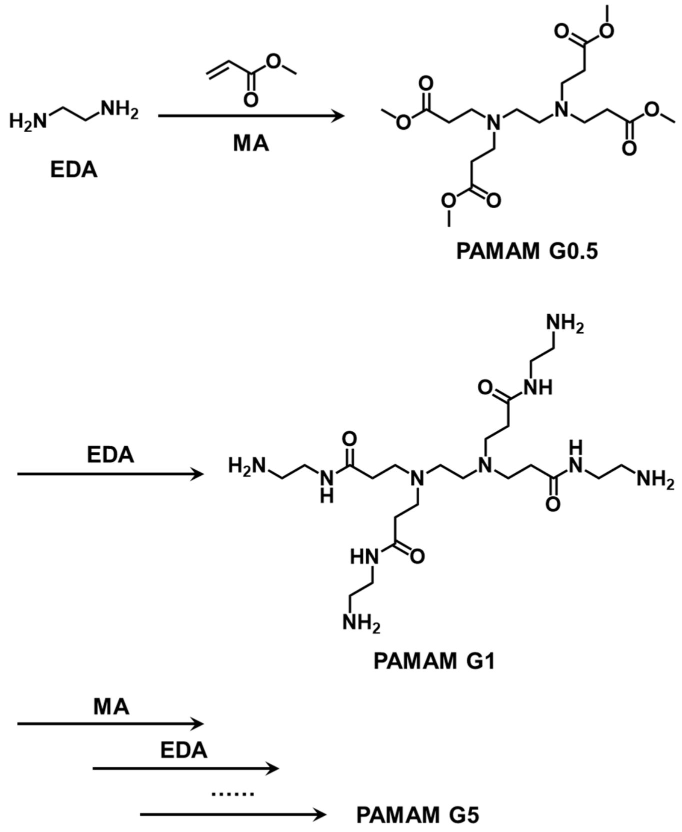

Figure S1. Reaction scheme of the PAMAM dendrimer.

Table S1 The molar ratio of the reactants for PAMAM preparation.

Sample	Molar ratio	
PAMAM G0.5	EDC : MA	= 1 : 4
PAMAM G1.0	PAMAM G0.5 : EDA	= 1 : 4
PAMAM G1.5	PAMAM G1.0 : MA	= 1 : 8
PAMAM G2.0	PAMAM G1.5 : EDA	= 1 : 8
PAMAM G2.5	PAMAM G2.0 : MA	= 1 : 16
PAMAM G3.0	PAMAM G2.5 : EDA	= 1 : 16
PAMAM G3.5	PAMAM G3.0 : MA	= 1 : 32
PAMAM G4.0	PAMAM G3.5 : EDA	= 1 : 32
PAMAM G4.5	PAMAM G4.0 : MA	= 1 : 64
PAMAM G5.0	PAMAM G4.5 : EDA	= 1 : 64

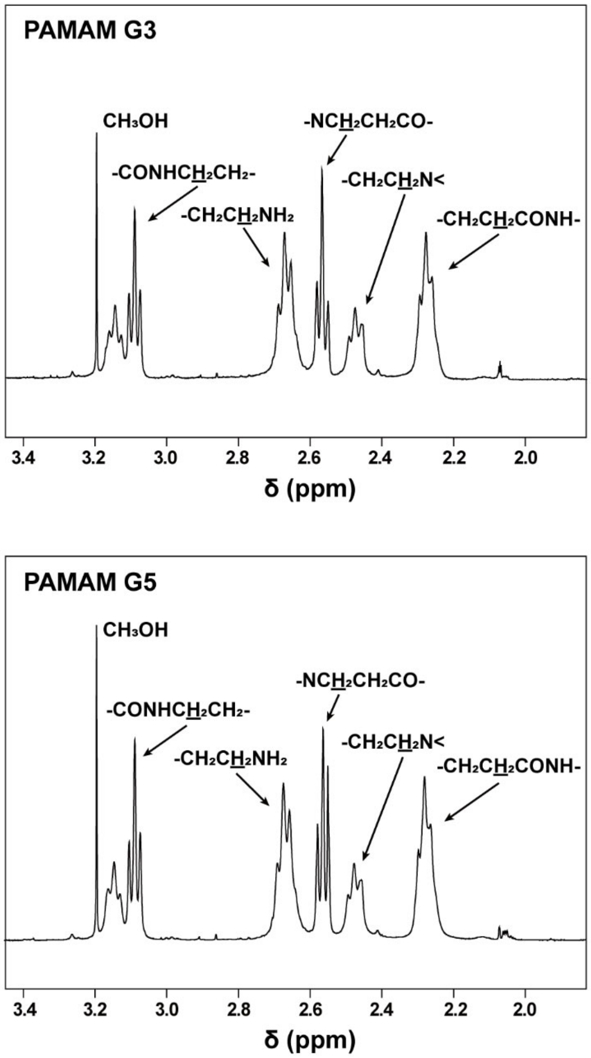

Figure S2. ^{1}H NMR spectra of PAMAM G3 and PAMAM G5.

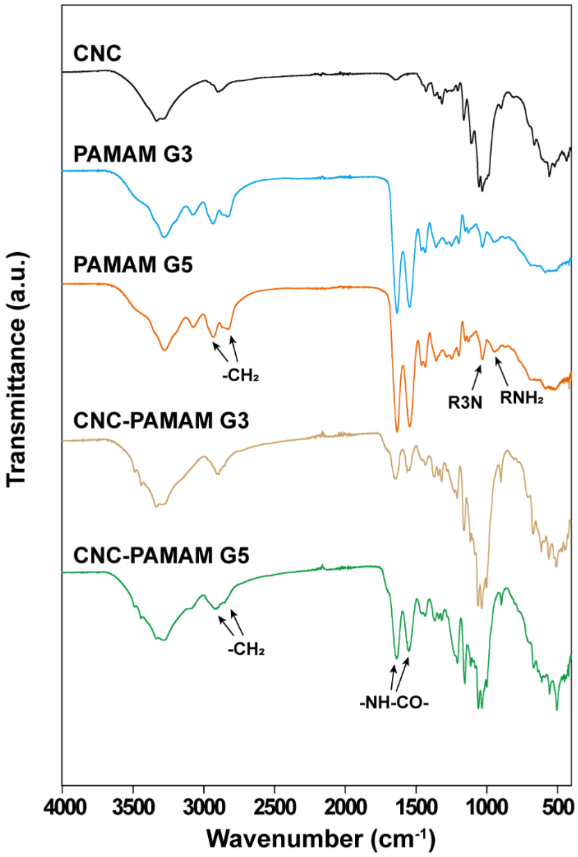

Figure S3. FTIR spectra of CNC, PAMAM G3, PAMAM G5, CNC-PAMAM G3 and CNC-PAMAM G5.
